# *Lacticaseibacillus paracasei* JS-3 Isolated from “Jiangshui” Ameliorates Hyperuricemia by Regulating Gut Microbiota and iTS Metabolism

**DOI:** 10.3390/foods13091371

**Published:** 2024-04-29

**Authors:** Jiahui Wu, Lvbu Aga, Leimengyuan Tang, Houxier Li, Nan Wang, Li Yang, Nan Zhang, Xiang Wang, Xueyong Wang

**Affiliations:** School of Chinese Meteria Medica, Beijing University of Chinese Medicine, Northeast Corner of Intersection of Sunshine South Street and Baiyang East Road, Fang-Shan District, Beijing 102488, China; wujiahui2017@126.com (J.W.); 15276201890@163.com (L.T.); 15928912991@163.com (H.L.); 18811385728@163.com (N.W.);

**Keywords:** gut microbiota, hyperuricemia, Jiangshui, *Lacticaseibacillus paracasei*, metabolomics, uric acid

## Abstract

**Background:** A diet high in purines can impair the function of the gut microbiota and disrupt purine metabolism, which is closely associated with the onset of hyperuricemia. Dietary regulation and intestinal health maintenance are key approaches for controlling uric acid (UA) levels. Investigating the impacts of fermented foods offers potential dietary interventions for managing hyperuricemia. **Methods:** In this study, we isolated a strain with potent UA-degrading capabilities from “Jiangshui”, a fermented food product from Gansu, China. We performed strain identification and assessed its probiotic potential. Hyperuricemic quails, induced by a high-purine diet, were used to assess the UA degradation capability of strain JS-3 by measuring UA levels in serum and feces. Additionally, the UA degradation pathways were elucidated through analyses of the gut microbiome and fecal metabolomics. **Results:** JS-3, identified as *Lacticaseibacillus paracasei*, was capable of eliminating 16.11% of uric acid (UA) within 72 h, rapidly proliferating and producing acid within 12 h, and surviving in the gastrointestinal tract. Using hyperuricemic quail models, we assessed JS-3’s UA degradation capacity. Two weeks after the administration of JS-3 (2 × 10^8^ cfu/d per quail), serum uric acid (SUA) levels significantly decreased to normal levels, and renal damage in quails was markedly improved. Concurrently, feces from the JS-3 group demonstrated a significant degradation of UA, achieving up to 49% within 24 h. *16S rRNA* sequencing revealed JS-3’s role in gut microbiota restoration by augmenting the probiotic community (*Bifidobacterium*, *Bacteroides unclassified_f-Lachnospiraceae*, and *norank_fynorank_o-Clostridia_UCG-014*) and diminishing the pathogenic bacteria (*Macrococus* and *Lactococcus*). Corresponding with the rise in short-chain fatty acid (SCFA)-producing bacteria, JS-3 significantly increased SCFA levels (*p* < 0.05, 0.01). Additionally, JS-3 ameliorated metabolic disturbances in hyperuricemic quails, influencing 26 abnormal metabolites predominantly linked to purine, tryptophan, and bile acid metabolism, thereby enhancing UA degradation and renal protection. **Conclusions:** For the first time, we isolated and identified an active probiotic strain, JS-3, from the “Jiangshui” in Gansu, used for the treatment of hyperuricemia. It modulates host–microbiome interactions, impacts the metabolome, enhances intestinal UA degradation, reduces levels of SUA and fecal UA, alleviates renal damage, and effectively treats hyperuricemia without causing gastrointestinal damage. In summary, JS-3 can serve as a probiotic with potential therapeutic value for the treatment of hyperuricemia.

## 1. Introduction

Uric acid (UA) is the byproduct of human purine metabolism [1]. Due to the lack of uricase, human UA levels (approximately 6.0 mg/dL) are much higher than other mammals (<2.0 mg/dL) [2,3]. Hyperuricemia is diagnosed when serum uric acid (SUA) exceeds 360 mmol/L in females and 420 mmol/L in males [4]. In recent years, with lifestyle and dietary changes, the foreign purine pool has increased, and the prevalence of hyperuricemia is escalating globally. For instance, a survey conducted in China revealed that the prevalence of hyperuricemia among Chinese adults increased significantly from 11.1% in 2015–2016 to 14.0% in 2018–2019 over a three-year period. Similarly, 20% of adults in the United States suffer from hyperuricemia [5,6]. Hyperuricemia has been recognized as a significant risk factor for the development of gout, renal dysfunction, hypertension, hyperlipidemia, diabetes, and obesity [7].

The etiology of hyperuricemia involves increased UA production, reduced renal excretion, or both [8]. When enzymes in the purine metabolism pathway are deficient, endogenous accumulation can occur, mainly from the lack of purine metabolism enzymes leading to an increase in the rate of nucleic acid decomposition and UA production, which is regulated by its own mechanism. The accumulation of UA can also be caused by the ingestion and exogenous absorption of purine-rich foods. Approximately two-thirds of UA is eliminated through the kidneys, with the remaining third degraded by intestinal microbiota [9]. UA transporters located in the intestinal epithelial cells facilitate the movement of UA from the bloodstream into the intestinal lumen, where it is either directly excreted or metabolized by gut microbiota. *Lactobacillus* and *Pseudomonas* species enhance the breakdown and elimination of UA in the intestine through the production of SCFAs [10]. However, foods rich in purines can greatly affect the effectiveness of gut microbiota, which may interfere with purine metabolism and increase SUA levels [11]. Therefore, controlling diet and maintaining intestinal health play crucial roles in managing UA levels.

Currently, pharmacotherapy remains the primary approach for managing hyperuricemia. Currently, common drugs for treating hyperuricemia include benzbromarone, probenecid, allopurinol, and febuxostat. They assist in the excretion of more uric acid by blocking renal transport proteins, or by inhibiting xanthine oxidase (XO) to reduce UA production. However, these treatments often cause liver, kidney, and gastrointestinal damage, and can sometimes be fatal [12,13,14,15]. These are just a few examples of drugs with specific side effects. Dietary management is another method for treating hyperuricemia. However, due to the difficulty in adhering to dietary restrictions over the long term, it may not be as effective as medication treatment. Currently, microbial remediation, as a cost-effective treatment method, has been widely accepted by the public due to its minimal bodily harm.

Substantial evidence supports the association of probiotics with various health benefits, including gastrointestinal regulation [16], the prevention and treatment of intestinal infections like ulcerative colitis [17], the modulation of intestinal immune function [18], the prevention and alleviation of allergic reactions [19], cardiovascular protection, etc. Recently, several strains of lactic acid bacteria that can utilize purines have been discovered, such as *Lactobacillus brevis DM9218*, which can reduce SUA levels in fructose-fed mice [20]. *Lactobacillus reuteri TSR332* and *Lactobacillus fermentus TSF331* reduced SUA levels in rats, and no significant side effects were observed during treatment [21]. *Lactobacillus fermentum 9-4* from Chinese fermented rice flour noodles might be a promising probiotic candidate for the development of low-purine foods [22]. However, the colonization ability and mechanism of action of probiotics in the intestine are different, and more sufficient evidence is needed to prove the impact of probiotics on hyperuricemia.

Probiotics, frequently used as dietary supplements, are incorporated into various products, predominantly fermented dairy goods [23]. Fermented foods are rich in lactic acid bacteria, which can produce organic acids to inhibit spoilage and pathogenic microorganisms. Jiangshui is a traditional fermented food from China’s northwest, predominantly containing lactic acid bacteria, acetic acid bacteria, and yeast, which are especially facultative anaerobic lactic acid bacteria that are important sources of probiotics. Probiotics in Jiangshui have shown UA degradation capabilities [24]. Therefore, the aim of this study is to screen and identify probiotics with UA degradation capabilities from “Jiangshui” in Gansu Province and conduct a probiotic viability assessment. Using a hyperuricemic quail model, the study explores JS-3’s ability to degrade UA and the degradation pathways involved.

## 2. Materials and Methods

### 2.1. Isolation of Strains with Uric Acid Degradation Ability

Traditional Jiangshui samples were purchased from Lanzhou, Gansu. Probiotics demonstrating UA-degrading capabilities were isolated from unprocessed Jiangshui samples utilizing a UA-specific selection medium (comprising 3 g/L KH_2_PO_4_, 17.1 g/L Na_2_HPO_4_·12H_2_O, 0.5 g/L NaCl, 0.5 g/L, MgSO_4_·7H_2_O, 2 g/L C_5_H_4_N_4_O_3_, 0.01 g/L CaCl_2_, and 12 g/L agar). A single colony was then transferred to a fresh culture medium, using UA as the sole carbon source, and incubated at 37 °C for 24 h. Subsequently, the isolated colony (JS-3) was streaked onto new MRS agar plates to procure a single colony, which was then subjected to three successive rounds of purification.

### 2.2. Determination of Uric Acid Degradation Ability of Cultured Strains In Vitro

To evaluate the ability to degrade UA, JS-3 was inoculated onto MRS medium, followed by anaerobic incubation at 37 °C for 24 h. Then, we centrifuged the bacterial solution at 664× *g* for 10 min, collected the strains, washed them twice with sterile water, and finally suspend the strains in the UA solution. Next, they were incubated at 37 °C for 24, 48, and 72 h. After cultivation, we centrifuged the bacterial solution at 900× *g* for 10 min and collected the supernatant, then added 20 μL HClO_4_ (0.1 mol/L) to the supernatant and use the UA assay kit (Biosino Biotechnolgy and Science Inc., Beijing, China) to detect changes in UA content before and after fermentation.

### 2.3. Strain Identification

The genomic DNA of the strain was obtained using the TIANamp bacterial DNA kit (Tiangen Biochemical Technology, Beijing, China). The PCR amplification, utilizing universal bacterial primers 27 F and 1492 R, comprised the following components: 25 µL of 2 × Pro Taq Master Mix, 1 µL of each primer, 20 µL of DNA template, and 3 µL of double-distilled water. The amplification protocol involved an initial denaturation at 94 °C for 30 s, followed by 35 cycles of denaturation at 98 °C for 10 s, annealing at 55 °C for 30 s, and extension at 72 °C for 1 min. The PCR product was sent to Sangon Biotech (Shanghai, China) Co., Ltd. for sequencing. Species were identified using the BLAST engine (NCBI) and the nucleotide sequence was submitted to GenBank for reference. The Gram staining technique was utilized to ascertain the bacterial species of JS-3, while scanning electron microscopy (SEM SU8100 N II Hitachi, Japan) facilitated the examination of JS-3’s morphology (Condition: Vacc = 3.0 kV, Mag = 15.0 k, WD = 10.9 mm).

### 2.4. Determination of Growth and pH Value of Bacterial Strains

The growth curve of the JS-3 strain was determined using the turbidimetric method [25]. The JS-3 strain was added to MRS medium at a 2% inoculation amount and cultured at 37 °C for 36 h. OD_600_ values were measured every 4 h by sampling and plotting the curve between the cultivation time and OD_600_ values. The sample was taken out every 2 h to measure its pH value and plot the curve of acidity over time during the fermentation process.

### 2.5. Animal Treatment

All experimental procedures and animal care were conducted by the guidance of the Animal Ethics Committee of Beijing University of Chinese Medicine (No. BUCM-4-2023010301-100). Seven-week-old male French quails (like humans, lacking uricase) were housed in 90 × 80 × 40 cm^3^ cages in the standard environment with temperature 25 ± 4 °C, air humidity 50–55%, and 12 h light/12 h dark cycle. After adaptive rearing, the quails were randomly divided into 4 groups: the standard control group (Control; drinking water), the model group (Model; drinking water), the benzbromarone-positive drug group (Ben; 20 mg/kg/d) and the JS-3 group (JS-3; 2 × 10^8^ cfu/d) for 2 weeks. The control group was fed with commercial formulation, while the other groups were fed the formulation with added 20% yeast extract powder (high-purine diet). Each group of animals was free to drink water.

### 2.6. Determination of UA Degradation by Quail Fecal Microbiota

We inoculated fresh quail feces into GAM medium, which were incubated anaerobically at 37 °C for 48 h, then transferred to new GAM where they continued to incubate for 48 h. After completion, we centrifuged at 4800× *g* for 10 min to obtain the gut microbiota of each group of quails. We washed the obtained bacterial community twice with sterile PBS solution, followed by centrifugation at 4800× *g* for 10 min and suspension in sterile PBS solution containing UA at 37 °C in an anaerobic incubator for 24 h. After cultivation, we used the UA detection kit (Biosino Biotechnolgy and Science Inc., Beijing, China) to detect the UA concentration in the supernatant.

### 2.7. 16S-rRNA Gene Sequencing

We extracted the total microbial community DNA from quail feces and amplified the variable region V3–V4 of the *16S rRNA* gene by PCR. Then, we constructed an Illumina Miseq library using purified amplicons. Sequencing was performed on the Miseq PE300/NovaSeq PE250 platform (Shanghai Majorbio Biopharmaceutical Technology Co., Ltd., Shanghai, China). Based on the overlap relationship, we connected the PE reads obtained from Miseq sequencing and controlled and filtered the sequence quality for bioinformatics analysis.

### 2.8. Detection of Short-Chain Fatty Acids (SCFAs) in Quail Feces

We detected SCFAs using gas chromatography (GC, Aglient 7890A, CA, USA). We accurately weighed 100 mg of fresh feces, added 1 mL of aqueous solution containing 2-ethylbutyric acid, adjusted the pH to 2–3 with formic acid, mixed well, allowed it to stand at room temperature for 20 min, centrifuged it at 7300× *g* at 4 °C for 15 min, collected the supernatant, and filtered it through a 0.22 μm microporous membrane. N_2_ served as the carrier, and the chromatographic column was DB-WAX (30 mm × 0.32 mm × 0.5 μm), with an FID detector and an injection volume of 1 μL for detection.

### 2.9. Non-Targeted Fecal Metabolomics Analysis

We accurately weighed 60 mg of fecal sample into an EP tube and added 600 μL of 80% aqueous methanol solution containing 0.01 mg/mL L-2-chlorophenylalanine (volume ratio: methanol: water = 4:1). This was pre-cool at −20 °C for 2 min, then ground for 2 min, sonicated for 10 min in an ice water bath, and allowed to stand at −20 °C for 30 min. Then, we centrifuged it at 4 °C and 10,625× *g* for 10 min, extracted the supernatant, filtered it, and placed it in an injection bottle. We used an equal volume of extraction solution from each sample and mixed it well to prepare a quality control sample (QC). Mass spectrometry conditions: ESI ion source and ion source temperature 350 °C. In ESI^+^ mode: ionization source voltage 4.0 kV, tube lens voltage 110 V, and capillary voltage 35 V. In ESI^−^ mode: ionization source voltage −3.0 kV, tube lens voltage −110 V, and capillary voltage −35 V. Collision voltage was 6–10 V. The flow rate of the sheath gas (Nz) was 40 arb, and the flow rate of the auxiliary gas (N_2_) was 20 arb, with a dry gas flow rate of 15 L/min and a scanning range of *m*/*z* 100~1500.

We used SIEVE (version 1.3, Thermo, Madison, WI, USA) and MetaboAnalyst 5.0 software to preprocess the raw data obtained. We analyzed compounds using the HMDB database combined with the Xcalibur 4.2 workstation to obtain the required metabolic molecules. Then, we imported fecal sample data from each group into SIMCA software (version 14.1), performed cluster analysis on the data using principal component analysis (PCA), and analyzed them using orthogonal partial least squares discriminant analysis (OPLS-DA). We used a variable importance in the projection (VIP) ≥ 1 and *p* < 0.05 as screening conditions combined with S-plots to screen metabolites and identify potential biomarkers.

### 2.10. Statistical Analysis

All experimental data were expressed as mean ± SD and statistically analyzed using SPSS 24.0 (SPSS, Chicago, IL, USA). Differences among multiple groups were demonstrated using One-way ANOVA. Wilcoxon tests with pairwise comparisons between groups were analyzed for non-parametric data. *p* < 0.05 was considered significant. All graphics were made in Graphid Prism 9 (GraphPad Software Inc., La Jolla, CA, USA).

## 3. Results

### 3.1. Screening, Isolation, and Identification of UA-Lowering Strains

**Figure 1:**
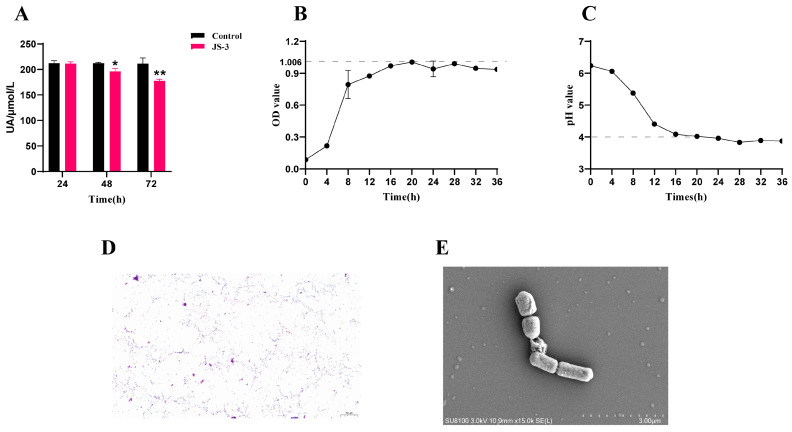
Screening and identification of JS-3 strain in Jiangshui. (**A**) The UA degradation ability of the JS-3 strain in vitro. (**B**) The growth curve of JS-3. (**C**) The acid production capacity of JS-3. (**D**) Gram stain of JS-3 (×400 magnification). (**E**) Scanning electron microscope morphology of JS-3. * *p* < 0.05, ** *p* < 0.01 compared with the control group. Data are shown as means ± SD (*n* = 8, each group).

To investigate whether the probiotics in Jiangshui have beneficial effects on hyperuricemia, a strain (JS-3) was isolated from commercial fermentation cultures with UA as the sole carbon and nitrogen source. JS-3 showed significant UA degradation, reducing levels by 16.11% within 72 h (Figure 1A). This strain had good growth characteristics and acid production ability. At 20 h, the growth of JS-3 reached its peak, and the pH value of the culture medium decreased by two orders of magnitude, subsequently transitioning to the decay phase (Figure 1B,C). Gram staining confirmed JS-3 as a G^+^ bacterium (Figure 1D). Compared to the NCBI BLAST database, the JS-3 strain was closely related to *Lacticaseibacillus paracasei*. Transmission electron microscopy (TEM) further validated JS-3’s typical morphology as consistent with *Lacticaseibacillus paracasei* (Figure 1E). These findings suggest that JS-3 is a promising probiotic for degrading UA.

### 3.2. JS-3 Decreases UA Level in Hyperuricemic Quails

**Figure 2:**
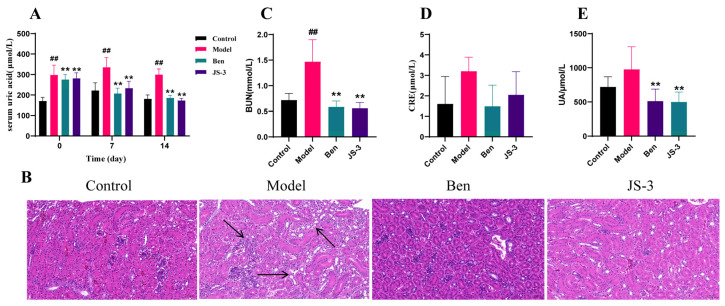
Effect of JS-3 on hyperuricemic quails. (**A**) The level of SUA. (**B**) Representative photomicrographs of H&E staining of quail kidney tissue (×400 magnification). (**C**,**D**) The levels of BUN and CRE. (**E**) The UA degradation ability of quail gut microbiota. ^#^ *p* < 0.05, ^##^ *p* < 0.01 compared with the control group; * *p* < 0.05, ** *p* < 0.01 compared with the model group. Data are shown as means ± SD (*n* = 8, each group). The arrow indicates the pathological damage to the kidney of the model quail.

Preliminary experiments have shown that JS-3 can degrade UA and survive in the gastrointestinal tract (Table S1). To further assess JS-3’s UA reduction efficacy in vivo, we selected quails that also lacked XO and established a hyperuricemia model. Compared to the control group, the SUA levels in the model group of quails were significantly increased. Following JS-3 intervention, the SUA levels significantly decreased, exhibiting a similar UA-lowering effect to the positive drug benzbromarone (Figure 2A). Notably, hyperuricemic quails showed significant kidney damage (reduced number of renal units, loose cytoplasmic staining of renal tubular epithelial cells, increased intercellular spaces, and vacuolar degeneration). JS-3 treatment significantly ameliorated these signs of UA-induced kidney damage (Figure 2B). Additionally, we measured the blood urea nitrogen (BUN) and creatinine (CRE) levels in each group, as depicted in Figure 2C,D. We observed a significant increase in BUN levels in the hyperuricemic quails, while the increase in CRE levels was not statistically significant. Post-treatment, the BUN levels in the JS-3 and Ben groups were significantly reduced, and CRE showed a decreasing trend, but there was no statistically significant difference. At the same time, quail feces were collected and cultured in GAM medium for 24 h, resulting in a 49% decrease in UA levels. The results showed that the gut microbiota of the JS-3 group possessed a notable capacity to degrade UA (Figure 2E). These findings suggest that JS-3 can effectively reduce UA levels in hyperuricemic quails and mitigate kidney damage, and that it is closely associated with gut microbiota, showing superior efficacy compared to the positive control drug, benzbromarone.

### 3.3. JS-3 Regulated UA-Induced Gut Microbiota Dysbiosis in Hyperuricemic Quails

**Figure 3:**
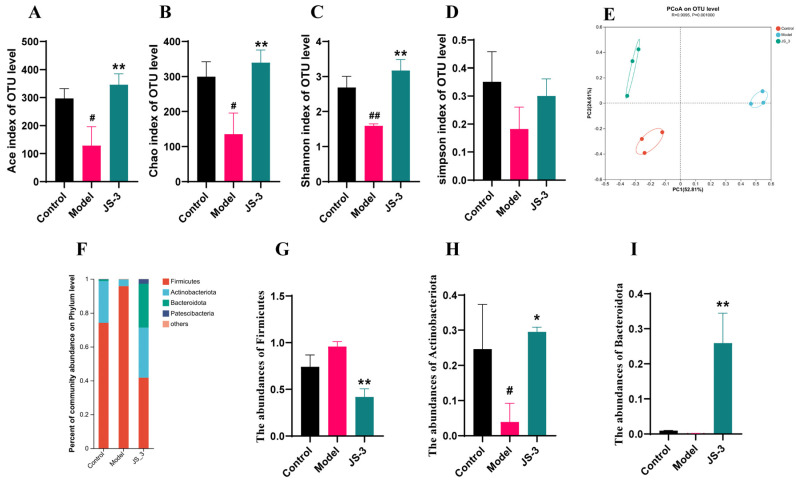
Effects of JS-3 treatment on the microbial diversity of gut microbiota in UA-exposed quails. (**A**–**D**) Microbial diversity analysis. (**E**) The weighted version of the UniFrac-based PCoA plots. (**F**) The gut microbiota composition at the phylum. The abundance of (**G**) Firmicutes, (**H**) Actinobacteriota, and (**I**) Bacteroidota. ^#^ *p* < 0.05, ^##^ *p* < 0.01 compared with the control group; * *p* < 0.05, ** *p* < 0.01 compared with the model group. Data are shown as means ± SD (*n* = 3, each group).

We performed a Miseq sequencing analysis on *16S rRNA* to determine the changes in gut microbiota associated with JS-3-treated hyperuricemic quails. The sequencing data obtained were reasonable and the sequencing depth covered rare new system types and most of the diversity (Figure S1A,B). The gut microbiota α-diversity in hyperuricemic quails significantly decreased, but following JS-3 intervention, its richness and diversity significantly increased (Figure 3A–D). Unweighted UniFrac-based principal coordinate analysis (PCoA) graphs were constructed to explore the similarities or differences in the overall community structure among the healthy, diseased, and treated groups. In the PCoA plot (Figure 3E), the control group and model group were separated along the PC1 and PC2 axes, indicating significant changes in the gut microbiota structure of the hyperuricemic quails. After the JS-3 intervention, the JS-3 group and the model group were significantly separated and tended to approach the control group, indicating a restoration of gut microbiota balance. Sequencing revealed that over 99% of the bacteria of the gut microbiota were divided into four phyla: Firmicutes, Actinobacteriota, Bacteroidota, and Patescibacteria, with Firmicutes and Actinobacteriota being predominant (Figure 3F). Compared with the control group, the abundance of Actinobacteriota was significantly lower and increased the abundance of Firmicutes in the model group. It is worth noting that JS-3 partially restored the abundance of these gut microbiota phyla and significantly increased the abundance of Actinobacteriota and Bacteroidota, while significantly reducing the abundance of Firmicutes (Figure 3G–I).

**Figure 4:**
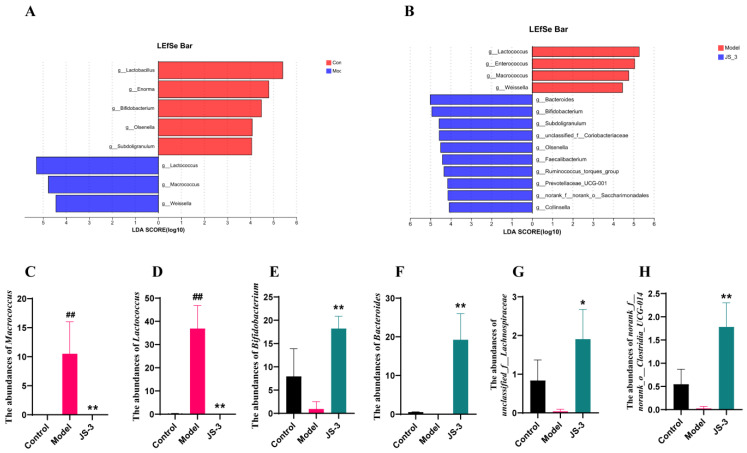
Effects of JS-3 treatment on the genus level of gut microbiota in UA-exposed quails. (**A**,**B**) Cladogram of the LEfSe results; Taxonomic represents statistically and biologically consistent differences. Only the taxa with a significant logarithmic LDA threshold score of >4 and *p* < 0.05 are shown. The abundance of (**C**) *Macrococus*, (**D**) *Lactococcus*, (**E**) *Bifidobacterium*, (**F**) *Bacteroides*, (**G**) *unclassified_f_Lachnospiraceae*, and (**H**) *norank_f_norank_o_Clostridia_UCG-014*. ^#^ *p* < 0.05, ^##^ *p* < 0.01 compared with the control group; * *p* < 0.05, ** *p* < 0.01 compared with the model group. Data are shown as means ± SD (*n* = 3, each group).

LEfSe analysis revealed 22 discriminative features (LDA > 4 and A *p* < 0.05). Among them, compared with the control group, the model group significantly reduced the abundance of *Lactobacillus*, *Enorma*, *Bifidobacterium*, *Olsenella*, and *Subdoliganulum*, and significantly increased the abundance of *Lactococcus*, *Macrococus*, and *Weissella* (Figure 4A). Post JS-3 intervention, there was a significant rise in the diversity at the genus level, including *Bacteroides*, *Bifidobacterium*, *Subdoligaranulum*, *unclassified_F_Coriobacteriaceae*, *Olsenella*, *Faecalibacterium*, *Ruminococcus_Torque_Group*, *Prevotellaceae_UCG-001*, *norank_F_Norank_O_Saccharimonadales*, and *Collinsella* (Figure 4B). We conducted a one-way ANOVA analysis of the gut microbiota and found that *Macrococcus* and *Lactococcus* were characteristic bacteria in the model group (Figure 4C,D), with a significant decrease after JS-3 intervention; *Bifidobacterium*, *Bacteroides*, *unclassified_f_Lachnospiraceae*, and *norank_f_norank_o_Clostridia_UCG-014* were characteristic bacteria of JS-3, which significantly increased after JS-3 intervention (Figure 4E–H). These results suggest that JS-3 regulates the gut microbiota structure of hyperuricemic quails, restores dysbiosis of the microbiota, enriches the diversity of the microbial structure, increases the abundance of beneficial bacteria, especially SCFA-producing bacteria, and reduces the abundance of harmful bacteria.

### 3.4. JS-3 Increased SCFA Levels in the Gut of Hyperuricemic Quails

**Figure 5:**
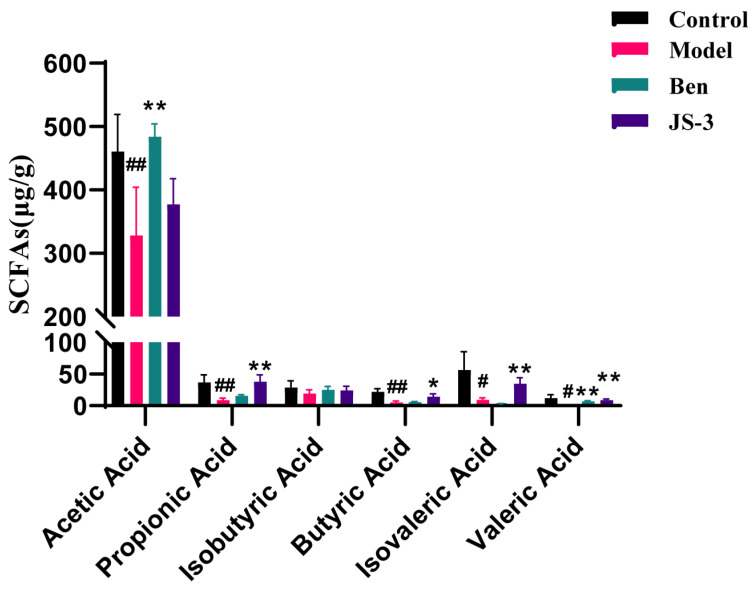
SCFA content of rats in each group. ^#^ *p* < 0.05, ^##^ *p* < 0.01 compared with the control group; * *p* < 0.05, ** *p* < 0.01 compared with the model group. Data are shown as means ± SD (*n* = 3, each group).

Following JS-3 treatment, SCFA-producing bacteria were markedly enriched and showed increased *Bifidobacterium*, *Lachnospiraceae*, and *Clostridia_UCG-014*. This indicates that the bacteria that produce SCFAs play an important role in the treatment of hyperuricemia with JS-3. In consideration of that, GC detected SCFAs in quail feces after two weeks of treatment. The results revealed that the levels of acetic acid, propionic acid, butyric acid, isovaleric acid, and valeric acid in the model group feces were significantly lower than those in the control group. Compared to the model group, JS-3 treatment significantly increased the concentrations of propionic acid, butyric acid, isovaleric acid, and isovaleric acid, and were superior to the positive drug benzbromarone (Figure 5).

### 3.5. Effect of JS-3 on the Fecal Metabolome

**Figure 6:**
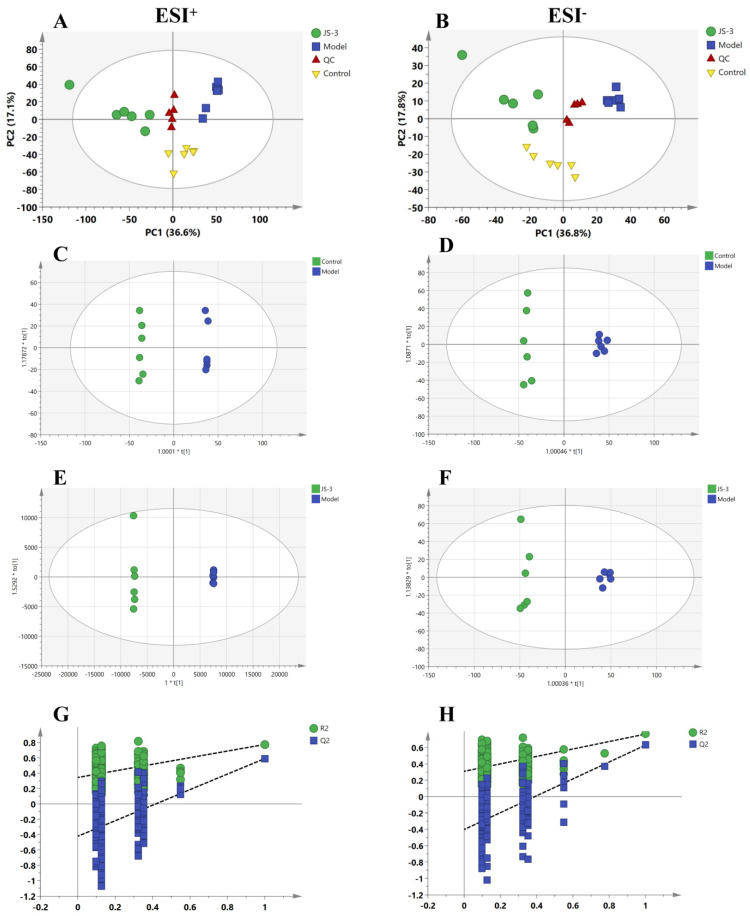
The PCA and OPLS-DA score plots of metabolic profiling. In positive mode and negative mode of (**A**,**B**), PCA score plot of each group. (**C**,**D**) OPLS-DA score plots of control group vs. model group; (**E**,**F**) JS-3 group vs. model group. (**G**,**H**) A total of 200 permutations were performed and plotted according to the resulting R2 and Q2 values (*n* = 6).

To investigate the mechanism of JS-3’s intestinal degradation of UA, we conducted a non-targeted metabolomics analysis on fecal samples. In the positive ion (ESI^+^) and negative ion (ESI^−^) mode, the PCA score plot showed that the dispersion points between the control group, model group, and JS-3 group quail feces were clearly distinguished, with good separability (ESI^+^ [R2X = 0.614, Q2 = 0.382] and ESI^−^ [R2X = 0.636, Q2 = 0.459]), reflecting significant changes in the normal fecal metabolism profile of hyperuricemic quails. JS-3 intervention improved the metabolic disorder of hyperuricemic quails to a certain extent (Figure 6A,B). To further investigate the biomarkers of significant changes in feces after JS-3 intervention, we compared the control group, model group, and JS-3 group using OPLS-DA. The OPLS-DA model (Figure 6C–F) showed that in ESI^+^ and ESI^-^ modes, the control group and JS-3 group were completely separated from the model group. In the permutation test, in ESI^+^ (R2 = 0.344, Q2 = −0.422) and ESI^-^ (R2 = 0.310, Q2 = −0.402) modes, it was shown that the model was not overfitting and was reliable (Figure 6G,H).

We combined the obtained data with the HMDB database to screen and identify differentially expressed metabolites using the VIP ≥ 1 and *p* < 0.05 criteria, as shown in Table S2. A total of 26 differential metabolites were screened, with 13 in both ESI^+^ and ESI^-^ modes. JS-3 treatment significantly modulated various metabolites compared to the model group, including an increase in xanthine, glutarylcarnitine, 4-(2-aminophenyl)-2,4-dioxobutanoic acid, alpha-muricholic acid, ursodeoxycholic acid 3-sulfate, and LysoPE (16:0/0:0), and a decrease in uric acid, 4,6-dihydroxyquinoline, p-cresol sulfate and sulfolithocholylglycine, implicating key metabolic pathways such as riboflavin, tryptophan, purine, and bile acid metabolism.

### 3.6. Correlations between Gut Microbiota, Metabolome, and Hyperuricemia-Related Parameters

**Figure 7:**
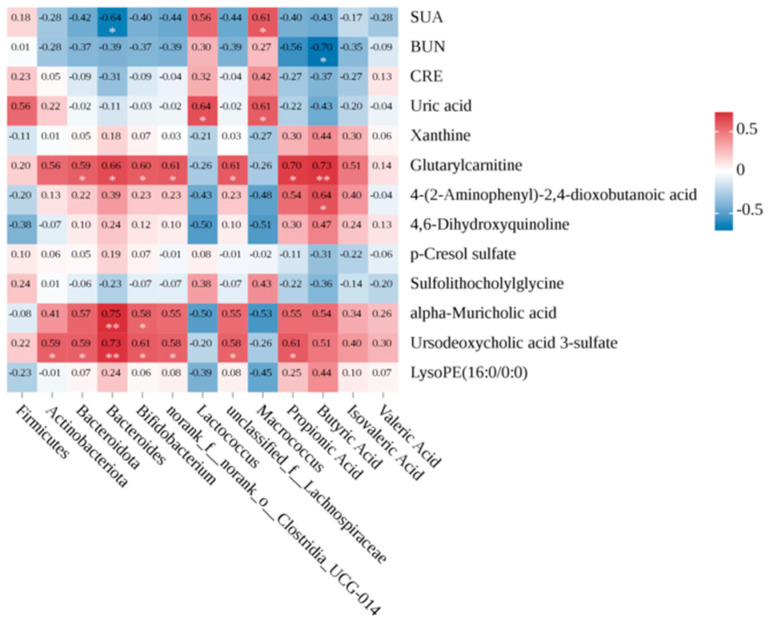
Associations of gut microbial changes with metabolome or hyperuricemia-related parameters. The r value is represented by the gradient color: red indicates positive correlation, and blue indicates negative correlation. * *p* < 0.05, ** *p* < 0.01.

We employed the Spearman correlation test to examine the relationships among gut microbiota composition, metabolomic profiles, and hyperuricemia-related parameters, as shown in Figure 7. The findings revealed that SUA levels were significantly positively correlated with *Macrococcus*, and significantly negatively correlated with *Bacteroides*. The analysis of SCFAs found a significant negative correlation, especially between butyric acid and BUN. We performed further analysis to associate gut microbiota with fecal differential metabolites, highlighting a predominant positive correlation with bile acid metabolites (ursodeoxycholic acid 3-sulfonate and alpha-muricholic acid) and purine metabolites (uric acid and glutarylcarbanitine). Notably, SCFAs, especially propionic acid and butyric acid, were significantly positively correlated with ursodeoxycholic acid 3-sulfonate, glutarylcarnitine, and 4-(2-aminophenyl)-2,4-dioxobutanoic acid. These correlations underscore the intricate interplay between intestinal microbial composition, metabolites, and host metabolic parameters.

## 4. Discussion

The prevalence of hyperuricemia has been on the rise, becoming increasingly common among younger populations, partly due to dietary changes. While medication remains a primary treatment, its preventive capabilities are limited, and side effects preclude long-term use. Therefore, it is necessary to explore new therapeutic approaches for hyperuricemia, and natural drugs and probiotics will be excellent choices for alternative therapies [26]. More and more probiotics are showing potential in UA degradation, host metabolism regulation, and chronic disease prevention with minimal adverse effects [21,27]. Jiangshui is a traditional, naturally fermented acidic condiment, whose distinctive flavor largely derives from the metabolic activity of microbial communities and their collective fermentation within the material system [28]. Consequently, the traditional fermentation of Jiangshui encompasses the synergistic actions of various microorganisms, such as lactic acid bacteria, yeasts, *Lactobacillus plantarum*, *Lactobacillus fermentum*, and *Candida* species [29,30,31,32]. Studies have shown that strains isolated from Jiangshui exert multiple beneficial physiological functions through the modulation of the intestinal microbiota, such as antioxidation, the alleviation of ulcerative colitis, the reduction of uric acid levels, and anti-anxiety effects, making them an important source of probiotics [24,33,34,35]. Therefore, this study attempted to screen and identify probiotics with UA-lowering capabilities from Gansu “Jiangshui”, providing candidate strains for the treatment of hyperuricemia.

This study isolated a strain with good UA degradation ability from Gansu “Jiangshui”, and further identified it as *Lacticaseibacillus paracasei* (JS-3). JS-3 has good growth characteristics, acid production ability, and can survive in the gastrointestinal tract. To assess JS-3’s UA degradation efficacy, in vivo experiments were conducted. Unlike most mammals, such as rats, mice, and rabbits, which contain uricase, humans and birds lack uricase, resulting in UA being the final product of purine metabolism [36]. Quails are used as experimental animals due to their suitable body size and superior reproductive performance. Despite the absence of a standardized hyperuricemia model for quails, we have adopted an appropriate hyperuricemia model based on a high-purine diet to better approximate clinical conditions. Our research findings indicate that, compared to the model group, both the groups treated with JS-3 and Ben for two weeks demonstrated a significant reduction in the levels of SUA and BUN in hyperuricemic quails, with JS-3 showing a superior effect. These findings were supported by a pathological slice analysis, including improvements in the watery degeneration of renal tubular epithelial cells, loose cytoplasmic staining, and increased intercellular space. However, the mechanism by which JS-3 reduces UA is still unclear.

It is established that approximately 70% of UA is excreted through the kidneys, while the rest is mainly excreted through feces or further metabolized by gut microbiota. Research has demonstrated that patients with hyperuricemia and gout have different gut microbiota structures and compositions compared to healthy individuals [10]. In our research, we collected feces from different groups of quails, incubating them in GAM medium to evaluate their UA degradation capacity. After 24 h, the UA levels in the JS-3 group decreased by 49%, suggesting that JS-3 has the potential to enhance intestinal metabolism and facilitate UA degradation. The beneficial effects of JS-3 on the intestines of hyperuricemic quails have attracted our interest in exploring the interactions between gut microbiota and metabolites.

Studies in the literature have demonstrated that the mechanism of reducing UA is intricately linked to metabolic disorders within the microbiota. The extensive application of probiotics has significant benefits in regulating the intestinal environment. For instance, *Lactobacillus brevis DM9218* has been shown to mitigate hyperuricemia by altering the disruption of gut microbiota and mucosal barrier function induced by fructose in mice models of the condition [20]. Our experiments revealed that the microbial diversity in the model group was significantly reduced compared to the control group. JS-3 restored the dysbiosis of gut microbiota in hyperuricemic quails, supported the restoration of healthy component structure, and significantly increased ecosystem diversity. Compared with the model group, after two weeks of JS-3 administration, Bacteroidota and Actinobacteriota significantly increased, and Firmicutes significantly decreased. *Macrococcus* and *Lactococcus* are characteristic bacteria of hyperuricemic quails, and JS-3 intervention significantly reduced them. Additionally, JS-3 markedly elevated the abundance of several beneficial bacteria (*Bifidobacterium*, *Bacteroides*, *unclassified_f-Lachnospiraceae*, and *norank_fynorank_o-Clostridia_UCG-014*). Consistent with these observations, the correlation analysis of gut microbiota and specific hyperuricemia-related parameters in our study also highlighted a positive correlation between the abundance of *Macrococcus* and *Lactococcus* with SUA and fecal UA levels, while the abundance of *Bacteroides* was negatively correlated with SUA. Notably, *Bacteroides xylanisolves* has been proven to be a possible pathway for treating goose hyperuricemia and gout [37].

Similar to the results of gut microbiota, fecal metabolomics analysis indicates that quails with hyperuricemia exhibit severe metabolic disorders. These metabolites and their derivatives, which can be excreted in feces or absorbed by the body, induce various biological effects. Owing to the remarkable acid production capability of JS-3 and its role in enriching SCFA-producing bacteria (*Bifidobacterium* [38], *Lachnospiraceae* [39], and *Clostridia_UCG-014* [40]), we initially measured the content of fecal SCFAs, which are one of the metabolites of gut microbiota. SCFAs play vital physiological roles, regulating the function of intestinal epithelial cells, maintaining the stability of the intestinal environment, and offering therapeutic benefits for conditions like intestinal inflammation [41]. Our findings indicated a substantial reduction in SCFA levels in hyperuricemic quails, which significantly improved following JS-3 treatment, especially affecting propionic acid, butyric acid, isovaleric acid, and isovaleric acid. Among them, butyric acid was significantly negatively correlated with BUN. According to reports, SCFAs, especially propionic acid and butyric acid, support ATP provision to intestinal wall cells, enhancing UA excretion [42,43]. For instance, rectal butyric acid administration has been reported to positively influence UA metabolism in healthy individuals [44]. Post JS-3 treatment, some abnormal metabolites were significantly reversed, such as purine metabolism products (uric acid, xanthine, and glutarylcarbanitine), tryptophan metabolism products (4-(2-aminophenyl)-2,4-dioxobutanoic acid and 4,6-dihydroxyquinoline), and bile acid metabolism products (sulfolithocholylglycine, alpha-muricholic acid, and ursodeoxycholic acid 3-sulfate). Among them, uric acid, ursodeoxycholic acid 3-sulfate, glutarylcarbanitine, and alpha-muricholic acid were closely related to gut microbiota. This indicates that changes in the gut environment affect similar metabolic pathways. The intervention of purine metabolism is especially crucial for the impact on hyperuricemia. Hyperuricemia is caused by an increase in UA production, a decrease in renal UA excretion, or a combination of both, and UA is a byproduct of purine metabolism that can be generated by xanthine. Following JS-3 intervention, there was a significant increase in the content of xanthine and a significant decrease in the content of UA, suggesting a reduction in UA synthesis and an enhancement in UA degradation. This prevents the accumulation of UA in the gut over a short period, which is beneficial for reducing the overall SUA levels in the body.

The kidney is a critical organ of the urinary system, playing a subsidiary role in the excretion of bile acids. Studies have shown that chronic renal failure can be accompanied by changes in bile acid balance. The kidney is also the main organ for UA excretion, and functional failure caused by hyperuricemia directly affects UA elimination. Our research results found that sulfolithocholylglycine is closely related to renal function, with a significant increase in sulfolithocholylglycine in high-dose Cd environments [45]. Moreover, p-cresol sulfate, a well-known intestinal-derived uremic toxin, contributes to kidney damage. P-cresol sulfate is usually excreted in urine through renal tubular secretions. However, in chronic kidney disease (CKD), p-cresol sulfate accumulates in the plasma and increases the risk of cardiovascular and renal disease development, leading to kidney injury and fibrosis [46]. Post JS-3 intervention, the levels of sulfolithocholylglycine and p-cresol sulfonate were significantly reduced, offering protection against kidney damage in hyperuricemic quails.

Alterations in bile acid metabolism, especially a decrease in secondary bile acid levels, are believed to contribute to the pro-inflammatory state. Among them, alpha-muricholic acid is strongly associated with a diminished severity of Crohn’s disease symptoms [47]. Furthermore, ursodeoxycholic acid 3-sulfatecan can suppress the overexpression of inflammasomes (especially NLRP6) in colitis mice, restore colonic mucus secretion in colitis mice, and are correlated with a heightened presence of *Bacteroidaceae* and *Clostridia_UCG-014* [48]. These findings align with our observations where, following JS-3 intervention, there was a significant increase in the content of alpha-muricholic acid and ursodeoxycholic acid 3-sulfate, and it was significantly positively correlated with the abundance of *Bifidobacterium*, *Bacteroides*, *unclassified_f_Lachnospiraceae*, *norank_f_norank_o_Clostridia_UCG-014*, and propionic acid.

The disruption of tryptophan metabolism plays a pivotal role in the development of hyperuricemia, a fact underscored by existing research [49]. 4-(2-aminophenyl)-2,4-dioxobutanoic acid and 4,6-dihydroxyquinoline are involved in tryptophan metabolism and closely related to intestinal inflammation. Studies indicate that IBS-QoL responders initially exhibit lower levels of 4-(2-aminophenyl)-2,4-dioxobutanoic acid and higher levels of 4,6-dihydroxyquinoline, noting substantial amelioration following probiotic intervention [50]. Aligning with our findings, JS-3 intervention markedly enhanced the content of 4-(2-aminophenyl)-2,4-dioxobutanoic acid, which was significantly positively correlated with butyric acid levels, and concurrently decreased the content of 4,6-dihydroxyquinoline. These results suggest that the selective modulation of specific types of gut microbiota systems, particularly those enriched with microbes involved in purine metabolism, tryptophan metabolism, and bile acid metabolism, may support the efficacy of JS-3 in alleviating host metabolic disorders, reducing UA synthesis, enhancing UA degradation, improving intestinal inflammation, and protecting against renal damage in hyperuricemic quails.

Despite our study’s efforts to elucidate the complex interplay between gut microbiota, fecal metabolomics, and hyperuricemia treatment via JS-3 therapy, several limitations persist. Firstly, while JS-3 can survive in the gastrointestinal tract, the colonization of JS-3 strains in gut microbiota has not yet been confirmed. Subsequent experiments can utilize PCR technology to detect the colonization of JS-3 in fecal samples. Secondly, due to the absence of intestinal transporter evaluation (such as urate transporter 1, glucose transporter 9), it is unclear how the beneficial effects of the intestine enter the body’s circulation, which should be the focus of our subsequent experiments. Finally, although the quail model of hyperuricemia closely mirrors human pathophysiology, further clinical research is essential to validate these findings.

## 5. Conclusions

For the first time, we extracted a candidate probiotic with the ability to degrade UA—*Lacticaseibacillus paracasei* (JS-3)—from Gansu “Jiangshui”. Using hyperuricemic quails as an animal model, the study investigated the UA degradation effects and pathways of JS-3. JS-3 can significantly reduce SUA and fecal UA levels in these quails, improve renal function, and regulate the structure and function of gut microbiota. While the interpretations of our findings should be approached with caution, the beneficial effects of JS-3 may be related to gut microbiota–host interactions. JS-3 restores microbial diversity and function in hyperuricemic quails, particularly the enrichment of SCFA-producing bacteria (*Bifidobacterium*, *Bacteroides*, *unclassified_f-Lachnospiraceae*, and *norank_f norank_o-Clostridia_UCG-014*) and the reduction of pathogenic bacteria (*Macrococcus* and *Lactococcus*). This in turn regulates metabolic imbalances primarily centered on SCFAs, purine, tryptophan, and bile acid metabolism. JS-3 can serve as a probiotic with potential therapeutic value for hyperuricemia, offering fresh perspectives for future research on the probiotic treatment of hyperuricemia.

## Data Availability

The original contributions presented in the study are included in the article/supplementary material, further inquiries can be directed to the corresponding author.

## Supplementary Materials

The following supporting information can be downloaded at: https://www.mdpi.com/article/10.3390/foods13091371/s1, Figure S1: Rarefaction curve (A) Sobs index, (B) Shannon index; Table S1: Survival rate of JS-3 in artificial gastric and intestinal fluids; Table S2: Potential biomarker information. The up (↑) and down (↓) arrows represent the relative increasing or decreasing trend of the metabolites.

## Abbreviations

UA, uric acid; SUA, serum uric acid; XO, xanthine oxidase; SCFAs, short chain fatty acids; PCA, principal component analysis; OPLS-DA, orthogonal partial least squares discriminant analysis; VIP, variable importance in the projection; TEM, transmission electron microscopy; BUN, blood urine nitrogen; CRE, creatinine; PCoA, principal co-ordinates analysis; CKD, chronic kidney disease.
