# Supplementary material for: Lacticaseibacillus paracasei JS-3 Isolated from “Jiangshui” Ameliorates Hyperuricemia by Regulating Gut Microbiota and iTS Metabolism
Source: Foods. 2024 Apr 29;13(9):1371. doi: 10.3390/foods13091371 (PMC11083236; doi:10.3390/foods13091371)
Supplement: Supplementary file 1 [file foods-13-01371-s001.zip › foods-2968563-supplementary/Supplementary data/Table S1.pdf]

Table S1 Survival rate of JS-3 in artificial gastric and intestinal fluids

simulated liquid	artificial gastric juice	simulated intestinal fluid	
	3h	4h	8h
PH=2	55.6±19.04	41.64±6.14	43.44±10.24
PH=3	92.06±7.58	99.28±5.05	98.68±2.71
PH=4	94.22±11.55	95.31±6.29	92.9±10.38
