# Supplementary material for: Lacticaseibacillus paracasei JS-3 Isolated from “Jiangshui” Ameliorates Hyperuricemia by Regulating Gut Microbiota and iTS Metabolism
Source: Foods. 2024 Apr 29;13(9):1371. doi: 10.3390/foods13091371 (PMC11083236; doi:10.3390/foods13091371)
Supplement: Supplementary file 1 [file foods-13-01371-s001.zip › foods-2968563-supplementary/Supplementary data/Table S2.pdf]

Table S2: Potential biomarker information. The up (↑) and down (↓) arrows represent the relative increasing or decreasing trend of the metabolites.

No.	**Rt/（min）**	***m/z***	**metabolites**	**Chemical formula**	**mode**	JS3 vs Model
1	0.69	381.07907	Dihydrocaffeic acid 3-O-glucuronide	$C_{15}H_{18}O_{10}$	$[M+Na]^+$	↓
2	0.737	118.08643	2-Pentenoic acid	$C_5H_8O_2$	$[M+H]^+$	↓
3	0.792	195.05084	Gulonic acid	$C_6H_{12}O_7$	$[M-H]^-$	↓
4	0.955	262.12878	O-Methylmalonylcarnitine	$C_{11}H_{19}NO_6$	$[M+H]^+$	↓
5	0.959	169.03571	Uric acid	$C_5H_4N_4O_3$	$[M+NH_4]^+$	↓
6	1.071	151.02625	Xanthine	$C_5H_4N_4O_2$	$[M-H]^-$	↑
7	1.422	276.14417	Glutarylcarnitine	$C_{12}H_{21}NO_6$	$[M+H]^+$	↑
8	1.859	434.18863	Acetophenazine	$C_{23}H_{29}N_3O_2S$	$[M+H]^+$	↓
9	6.161	162.056	4-(3-Pyridyl)-3-butenoic acid	$C_9H_9NO_2$	$[M-H]^-$	↑
10	6.161	206.04559	4-(2-Aminophenyl)-2,4-dioxobutanoic acid	$C_{10}H_9NO_4$	$[M-H]^-$	↑
11	6.216	162.05493	4,6-Dihydroxyquinoline	$C_9H_7NO_2$	$[M+H]^+$	↓
12	7.156	187.00702	p-Cresol sulfate	$C_7H_8O_4S$	$[M-H]^-$	↓
13	7.458	493.27911	Erinacine P	$C_{27}H_{40}O_8$	$[M+H]^+$	↓
14	9.776	381.19144	12-Oxo-20-trihydroxy-leukotriene B4	$C_{20}H_{30}O_7$	$[M-H]^-$	↑
15	9.783	464.33389	3-Hydroxy-9Z-octadecenoylcarnitine	$C_{25}H_{47}NO_5$	$[M+H]^+$	↓
16	9.8	589.31134	25-Cinnamoyl-vulgaroside	$C_{34}H_{46}O_7$	$[M+H]^+$	↑
17	10.437	503.26459	Withaperuvin B	$C_{28}H_{38}O_8$	$[M+Na]^+$	↑
18	10.536	518.27454	Travoprost	$C_{26}H_{35}F_3O_6$	$[M+Na]^+$	↑
19	12.077	462.2673	desmethylastemizole	$C_{27}H_{29}FN_4O$	$[M+NH_4]^+$	↓
20	12.121	512.26813	Sulfolithocholylglycine	$C_{26}H_{43}NO_7S$	$[M-H]^-$	↓

No.	**Rt/（min）**	***m/z***	**metabolites**	**Chemical formula**	**mode**	JS3 vs Model
21	12.207	514.28308	Taurallocholic acid	$C_{26}H_{45}NO_7S$	$[M-H]^-$	↓
22	14.394	407.27982	alpha-Muricholic acid	$C_{24}H_{40}O_5$	$[M-H]^-$	↑
23	14.551	471.24176	Ursodeoxycholic acid 3-sulfate	$C_{24}H_{40}O_7S$	$[M-H]^-$	↑
24	16.921	595.38379	Asparagoside B	$C_{33}H_{56}O_9$	$[M-H]^-$	↓
25	17.475	438.26227	Dodecanoyl-sn-glycero-3-phosphocholine	$C_{20}H_{42}NO_7P$	$[M-H]^-$	↑
26	18.44	452.27808	LysoPE(16:0/0:0)	$C_{21}H_{44}NO_7P$	$[M-H]^-$	↑
