# Supplementary figures and images for: Lacticaseibacillus paracasei JS-3 Isolated from “Jiangshui” Ameliorates Hyperuricemia by Regulating Gut Microbiota and iTS Metabolism
Source: Foods. 2024 Apr 29;13(9):1371. doi: 10.3390/foods13091371 (PMC11083236; doi:10.3390/foods13091371)

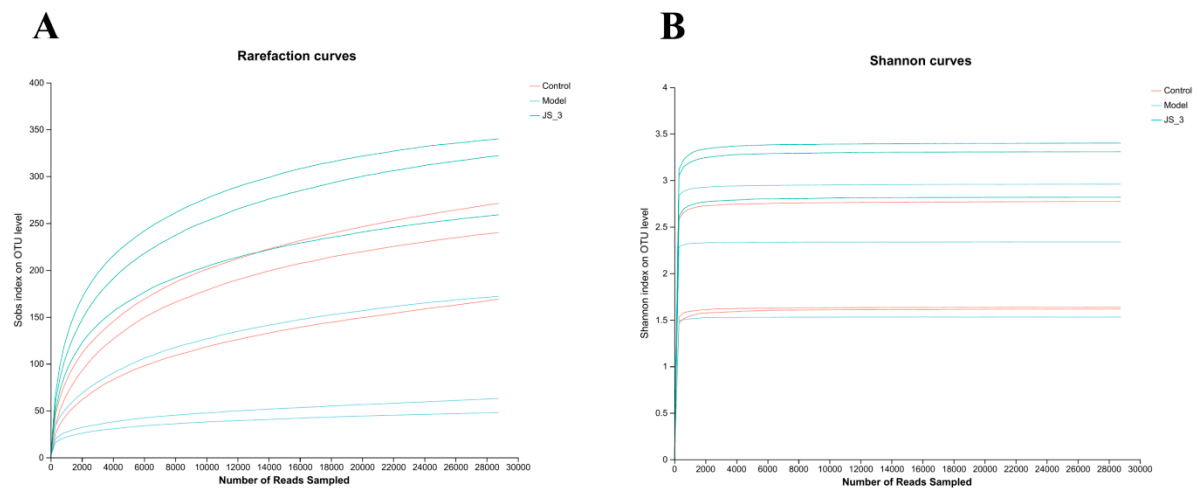

Fig. S1. Rarefaction curve (A) Sobs index, (B) Shannon index.

Supplement: Supplementary file 1 [file foods-13-01371-s001.zip › foods-2968563-supplementary/Supplementary data/Fig. S1.pdf]

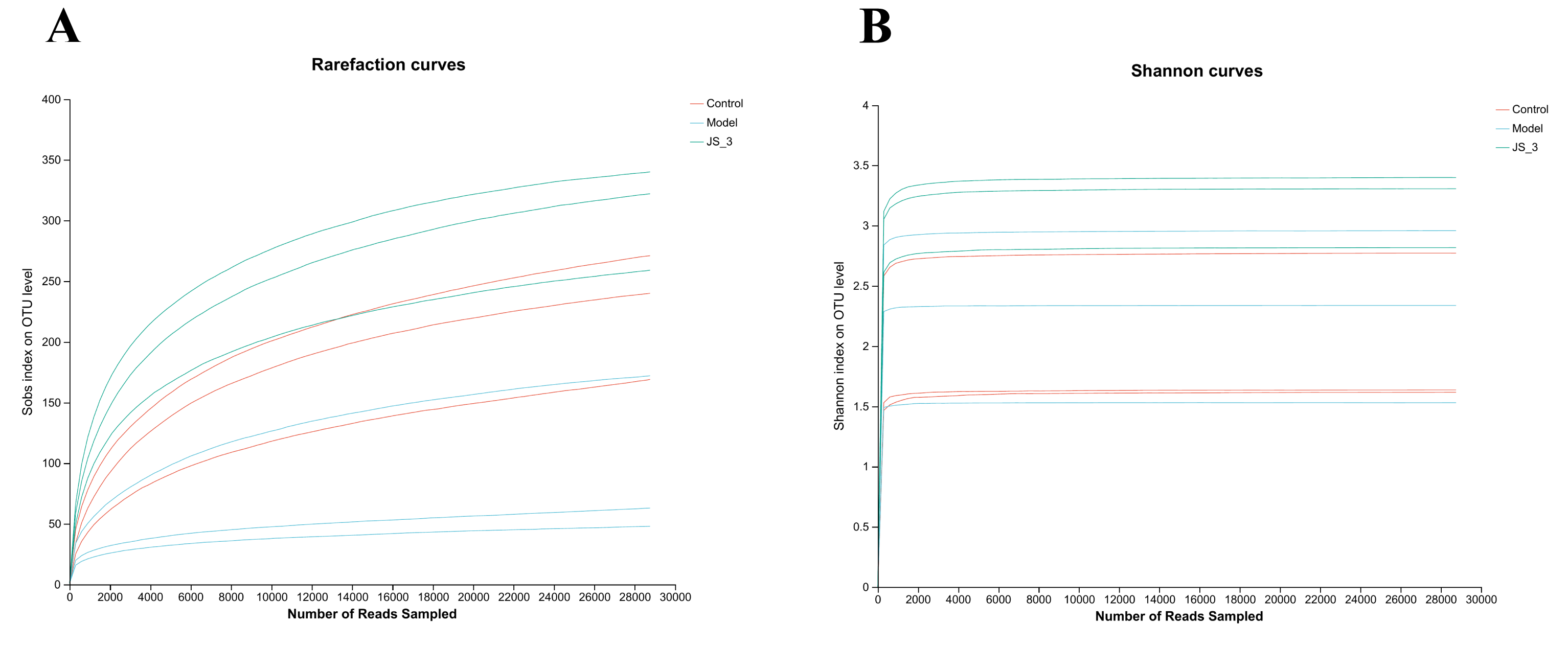

Supplement: Supplementary file 1 [file foods-13-01371-s001.zip › foods-2968563-supplementary/Supplementary data/Fig. S1.tif]
